# Drive-through Medicine for COVID-19 and Future Pandemics

**DOI:** 10.5811/westjem.2020.9.48799

**Published:** 2020-12-16

**Authors:** Jessica Ngo, Shashank Ravi, Naryeong Kim, Milana Boukhman

**Affiliations:** * VA Palo Alto Healthcare System, Department of Emergency Medicine, Palo Alto, California; † Stanford University School of Medicine, Department of Emergency Medicine, Stanford, California; ‡ Stanford University, Stanford, California

## BACKGROUND

The outbreak of coronavirus disease 2019 (COVID-19), caused by severe acute respiratory syndrome coronavirus 2 (SARS-CoV-2), has reached pandemic levels and continues to spread across much of the world.^1^ Hospitals and public health authorities are struggling to appropriately manage potentially infectious individuals to limit transmission to others, care for ill patients with proven or suspected COVID-19, and restart society after the pandemic. Crucial features of a successful response to a pandemic virus are early detection and isolation of potentially infectious individuals.^2^

With the increase in the global population, estimated to reach 9.7 billion by 2050, public health systems have less time to detect and contain a pandemic before it spreads. Fast and efficient screening and testing are key tools in controlling pandemics.^3^ This capacity should be rapidly scalable. During the current pandemic, most of the testing in the United States has occurred in emergency departments (ED), hospitals, and clinics. More recently, stand-alone diagnostic centers have been used as well, primarily by local public health departments. At the Stanford ED, there was a surge of patients wanting to be tested, which created a need for an efficient screening and testing model for COVID-19.

Testing and using drive-through models after appropriate triage have been shown to be more efficient compared to testing offered in traditional medical settings.^4^ Drive-through medicine also offers additional benefits, including better infection control and resource allocation.^5^ Furthermore, these models can also be rapidly scaled up to provide vaccinations and dispense medications.^6^ Therefore, it is important to consider drive-through medicine as a tool in controlling pandemics. Here we examine best practices of drive-through medicine based on global and US experiences.

## RISK OF TRANSMISSION

COVID-19 as well as a number of other coronaviruses have been shown to be transmitted by three main epidemiological patterns: family clusters; healthcare-acquired infections; and community cases.^7^,^8^ Hospitals, enclosed housing complexes, religious complexes, and mass transportation are documented sites of super-spreader events for SARS, Middle East respiratory syndrome, and COVID-19.^5^,^9^,^10^ The implementation of drive-through medicine decreases the risk of super-spreader events by reducing transmission between patients as well as workers in an already outstretched system.

COVID-19 is transmitted through respiratory droplets as well as through airborne mechanisms. A simulation of a drive-through model for an influenza pandemic demonstrated a social distancing strategy that reduced the risk of infection between patients and workers.^11^ Standard operating procedures for most EDs is to direct patients to potentially crowded waiting areas, which increases the risk of cross-infection, especially for respiratory illnesses. The drive-through influenza clinic simulation allowed patients to stay in their vehicles as healthcare workers evaluated them. The study found that the drive-through model was a feasible alternative that provided a social distancing strategy by using the patient’s vehicle as an *isolation compartment*.^7^ Furthermore, the stationing of the drive-through model outdoors reduced contamination of inpatient spaces.

The number of negative pressure rooms and high efficiency particulate air (HEPA)-filtered rooms that are appropriate to treat patients who are potentially infected by airborne pathogens is limited. Drive-through medicine can be a rapidly scalable solution to build capacity for evaluating and testing such patients safely. Furthermore, medical staff may feel more protected if potentially infectious individuals do not enter healthcare settings, thus reducing absenteeism previously observed in pandemics.^12^

## ALLOCATION OF RESOURCES

The COVID-19 pandemic is placing additional strain on a number of already overstretched healthcare facilities. In EDs, the surge in patients has threatened to exacerbate crowding. The World Health Organization estimates that 15–35% of the population will develop an influenza-like illness during a typical pandemic. In the US, this could result in an estimated 18–42 million ED visits, exacerbating existing ED crowding.^13^ The Institute of Medicine has called ED crowding a national threat because it would diminish regional disaster response capacity.^14^ Furthermore, during pandemics and epidemics, the demand for critical care services may quickly exceed available intensive care unit staff, beds, negative pressure rooms, and equipment, leaving a large infected population without lifesaving critical care.^15^ Efficient methods of testing and screening patients with influenza-like illness would expand the limited surge capacity of our healthcare system.

Drive-through settings allow for mass evaluation and testing in a quick, efficient manner while limiting exposure to healthcare workers and conserving personal protective equipment (PPE). In a typical healthcare setting, all involved hospital staff would have to change PPE after examining one potentially infectious patient. In some drive-through models, only the medical staff in the specimen-collection station would need to change disposable apron gowns and gloves without changing the entire PPE (inner and outer gloves, N95 respirator, eye-shield/face shield/goggles, and hooded gowns).^18^

Rooms do not need to be cleaned in between patients, preventing further potential exposure of house cleaning staff and reducing turnaround time between patients. This then opens up beds in the ED for other patients to be seen. In the drive-through influenza clinic simulation, the physicians transitioned to the next vehicle without waiting for the previous one to be discharged from the drive-through model.^11^ The patient’s vehicle acted as a moving examination room that relieved the need for fixed rooms and spaces, as in traditional healthcare settings.^11^

Time is also an essential resource during pandemics. Drive-throughs have been shown to reduce throughput times compared with care provided in traditional medical settings for both testing and vaccination. A study that measured the feasibility of influenza vaccinations for children in a drive-through clinic setting found that the median total clinic time regardless of services was nine minutes.^16^ In another study that focused on throughput times for adults and children during drive-through influenza vaccinations, the median throughput time was five minutes. The simulation concluded that drive-through vaccination clinics could rapidly vaccinate large populations of children and adults.^17^ In South Korea it takes an hour to test each patient in traditional healthcare settings, but in drive-throughs the time is reduced to 10 minutes per patient.^14^ The implementation of drive-throughs in South Korea has reduced the strain on resources during the COVID-19 pandemic and has been critical in contributing to South Korean success in curbing the spread of COVID-19.^13^

In a traditional healthcare setting, in addition to more expansive cleaning of surfaces, an airborne infection isolation room would require 12 air changes per hour, which could add 30 minutes or more turn-around time between patients.^18^ A drive-through system, however, does not require such cleaning procedures as the testees’ cars can be used as specimen-collection rooms. This means that fewer medical and cleaning staff are necessary.

Additionally, using drive-throughs can potentially be a more cost-effective method of providing screening and testing compared to an ED or clinic. PPE costs for the Stanford ED were calculated at $26 per patient for a physician and nurse team who would normally interact with the patient. Drive-throughs can be staffed without any additional direct patient-care labor costs by repurposing nurses who would otherwise be providing triage or care in an ED setting. The primary additional labor cost in the below model is a facilities traffic coordinator.

## STANFORD EXPERIENCE AND BEST DRIVE-THROUGH MODEL PRACTICES

Drive-through systems have been implemented in various settings during the COVID-19 pandemic. By adding the capacity to run drive-through evaluation, testing, and vaccination to EDs, outpatient clinics, and community testing sites/centers, there has been a global effort to diagnose and control COVID-19 more efficiently.

### Stanford Experience

At Stanford Health Care, a multifocal approach was used for COVID-19 testing of patients who did not require hospitalization. In the ED, patients who arrived with respiratory complaints were screened upon arrival to the department. Based on risk factors including age, comorbidities, and vital signs at presentation, patients were either triaged into the main ED or sent to an outdoor testing area. If they presented to the triage area in their vehicle, nurses obtained a quick history and vital signs check with the patient still in the vehicle, and if appropriate, patients were routed to a garage while still remaining in their vehicle. If they presented on foot, they were directed to a section of the garage with chairs after the triage process.

A medical screening exam was then performed by a physician via telemedicine, which determined whether the patient met the criteria for a COVID-19 test. Nasopharyngeal swabs were obtained, and patients were discharged pending test results, which were subsequently communicated to patients by call-back nurses and Stanford’s healthcare web application. The ED provides unscheduled care, and therefore efficiency and throughput are important metrics for any care process. The drive-through testing model was present in the ED for four weeks, from March 13, 2020–April 8, 2020. During this time, a total of 790 patients were screened, with 48 of those patients, or 6.1%, ultimately requiring an actual in-person ED visit for treatment. Length of stay for patients who presented to the ED needing a COVID-19 test and could be discharged decreased from 1.27 hours prior to drive-through implementation to 0.38 hours post drive-through implementation. The positivity rate of COVID-19 for patients screened in the ED drive-through site was 8.61%.

The ambulatory clinics at Stanford Health Care also used a scheduled drive-through model, with screening for patients being performed via telemedicine visits while the patient was still at home. If the patient screened into being tested, they were then given a dedicated time to arrive by vehicle to one of several outpatient, outdoor testing sites set up throughout the catchment area. These testing options helped to promote PPE conservation and healthcare worker safety.

### Automation, Efficiency and Safety Through the Use of Digital Health and Telemedicine

Numerous other medical centers, as well as California public health departments, have initiated drive-through testing and evaluation programs.^19^,^20^ In the US, the use of telemedicine in these drive-through systems is especially notable. Telemedicine reduces unnecessary contact between physicians and patients and develops standard protocols of screening via online or telephone visits.^19^ Alphabet’s Verily health sciences testing initiative, contracted by the California Health Department, deployed stand-alone testing sites and tested more than 3700 cases.^20^ By using community-testing models with online screening and online scheduling, Verily generates order automatically without physicians needing to see the patient. This promotes the safety of healthcare workers, while automating significant portions of the entire process.^20^

### Adapting Drive-Through Models to Specific Settings and Resources

In designing drive-through evaluation and testing models, adaptability and fit are key. For example, phone screenings may be particularly relevant in communities where patients have limited internet access or where this type of workflow is better suited for resources available to local public health departments. Palm Beach County, Florida, offers drive-through testing, but patients are screened over the phone in order to get tested. The Healthcare District of Palm Beach County is handling phone screenings, scheduling appointments, and conducting tests with the Florida Army National Guard and Palm Beach County’s Department of Emergency Management and the Governor’s Office.^19^

Internationally, South Korea’s implementation of drive-through systems has proven to be effective in reducing transmission and allocating resources efficiently. The first drive-through system in South Korea for COVID-19 was implemented in February 2020.^14^ Since then, 68 screening centers among the 577 centers in the county have established drive-throughs for evaluation and testing. The drive-through operations are standardized across these centers and include four stations: registration and questionnaire; examination; specimen collection; and instructions and information leaflet.^14^

### Utilization of Drive-Throughs for Evaluation, Testing, Vaccinations and Dispending of Medications

Although during the current pandemic the drive-throughs have been used primarily for screening of patients with influenza-like illness and for testing, as the pandemic and our response to it evolves, the same models could also be used for vaccinations and dispensing of medications. The Hawaii Department of Health developed a similar drive-through model for dispensing Strategic National Stockpile medication.^21^ During the two-hour session in April 2005, 622 patients were evaluated with a rate of 5.2 persons per minute with minimal human contact. The results found that local health departments, particularly in rural areas, could facilitate healthcare services and limit mortality during a public health emergency when dispensing medication. This model also demonstrates that drive-throughs are effective in both rural and urban areas for both testing and outpatient treatment. Drive-through medicine has the value of rapid scalability of capacity and services provided as the stresses on individual health systems and communities vary.

## POTENTIAL CHALLENGES AND SOLUTIONS

Through the previous implementation of drive-throughs, there have been several limitations and challenges identified. At Stanford, we experienced firsthand that drive-through testing centers may be impractical in certain outdoor conditions, particularly in areas with colder or damp climates. We mitigated this challenge by having the drive-through testing in a parking garage and through the use of heat lamps during periods of colder weather. When drive-throughs were conducted in garages, a Stanford study found carbon monoxide levels to be safe.^7^ Another option would be to use negative pressure tents for triage or additional ED space^22^ or tents with HEPA filters.^23^ Coordination between extensive entities within our health system was needed for initial activation, including security, facilities, information technology, and parking services. State and county department of public health permits also had to be obtained for new treatment areas.

Another challenge at Stanford was an initial lack of standardization around registration, charting, and providing discharge instructions. We used technology including secure text communication to improve communication among the clinical and non-clinical staff. Standardization in the electronic health record also facilitated keeping up with medical charting, appropriate symptomatic test ordering, and discharging a high throughput volume of patients with appropriate discharge instructions. Staff allocation of nurses and providers was an ongoing challenge in a health system with limited staff resources, requiring a daily review of optimization of each care area inside and outside the ED.

Outside of Stanford, there was observed to be a lower barrier for access to testing in drive-through centers, which may be both an advantage as far as improving access, but also a limitation. In South Korea, people were visiting drive-through testing centers for unnecessary repeat tests, which can be problematic from the standpoint of resource allocation due to the costs of testing as well as limited testing supplies. Sentara Healthcare and M Health Fairview suspended drive-through centers to preserve a limited supply of testing supplies (PPE, COVID-19 test kits).^24^ This potential limitation could be mitigated by educating the public on proper testing criteria and prior screening, either online or in-person.

Moreover, access to higher level medical care is not available in stand-alone testing centers, which may result in suboptimal medical care of patients in such centers. For example, in South Korea implementation of testing centers meant that immediate response to medically unstable patients could be limited if drive-through testing centers are located far from emergency centers and hospitals. Therefore, appropriate prior triage is critical, and sicker patients should be referred to testing at medical centers with appropriate level of care. Thus, these issues can be mitigated by appropriate triage and patient communication.

By addressing these limitations, best practices of drive-through medicine can be implemented across states that are currently highly impacted by the COVID-19 pandemic.

## CONCLUSION

Rapid testing has been a key tool in helping to control COVID-19 and other pandemics.^3^ Drive-through medicine has been used for provision of testing during the COVID-19 pandemic and has been shown to offer a number of advantages including rapid scalability, safety, faster throughput times, and efficient allocation of resources. Furthermore, as a pandemic evolves, drive-through medicine can be potentially used for vaccinations and dispensing of medications, if medically appropriate. EDs, clinics, medical centers, and public health departments should consider utilization of drive-through models as a tool in controlling the COVID-19 pandemic.
